# Genetic and Environmental Influences on Retinopathy of Prematurity

**DOI:** 10.1155/2015/764159

**Published:** 2015-05-21

**Authors:** J. M. Ortega-Molina, R. Anaya-Alaminos, J. Uberos-Fernández, A. Solans-Pérez de Larraya, M. J. Chaves-Samaniego, A. Salgado-Miranda, R. Piñar-Molina, A. Jerez-Calero, J. L. García-Serrano

**Affiliations:** ^1^Department of Ophthalmology, San Cecilio University Hospital, Dr. Oloriz Street No. 16, 18012 Granada, Spain; ^2^Department of Paediatrics, San Cecilio University Hospital, Dr. Oloriz Street No. 16, 18012 Granada, Spain; ^3^Retinopathy of Prematurity Programme, San Cecilio University Hospital, Dr. Oloriz Street No. 16, 18012 Granada, Spain

## Abstract

*Objective*. The goals were to isolate and study the genetic susceptibility to retinopathy of prematurity (ROP), as well as the gene-environment interaction established in this disease. *Methods*. A retrospective study (2000–2014) was performed about the heritability of retinopathy of prematurity in 257 infants who were born at a gestational age of ≤32 weeks. The ROP was studied and treated by a single pediatric ophthalmologist. A binary logistic regression analysis was completed between the presence or absence of ROP and the predictor variables. *Results*. Data obtained from 38 monozygotic twins, 66 dizygotic twins, and 153 of simple birth were analyzed. The clinical features of the cohorts of monozygotic and dizygotic twins were not significantly different. Genetic factors represented 72.8% of the variability in the stage of ROP, environmental factors 23.08%, and random factors 4.12%. The environmental variables representing the highest risk of ROP were the number of days of tracheal intubation (*p* < 0.001), postnatal weight gain (*p* = 0.001), and development of sepsis (*p* = 0.0014). *Conclusion*. The heritability of ROP was found to be 0.73. The environmental factors regulate and modify the expression of the genetic code.

## 1. Introduction

Retinopathy of prematurity (ROP) is a consequence of an arrest in normal retinal neural and vascular development, which determines the aberrant retinal regeneration [1, 2]. Several factors might indicate the existence of a hereditary component of ROP: (a) the differences in the incidence of ROP between different ethnic groups [3]; (b) an uneven progression of ROP despite the presence of common environmental factors [4–7]; (c) and the correlation between the progression of the retinal vascularization and the gestational age [8].

Twin studies show that from 70% to 80% of the susceptibility to ROP is conditioned by genetic factors [9, 10]. In premature infants, the phenotype of ROP, therefore, will be the result of the joint action of genetic and environmental factors and their interaction [11, 12].

The objective of our study was to analyze the heritability of ROP and the contribution of different environmental risk factors in three cohorts: monozygotic twins, dizygotic twins, and premature infants of simple birth.

## 2. Subjects, Material, and Methods

A total of 257 premature infants, 153 of simple births, 66 dizygotic twins, and 38 monozygotic twins were studied retrospectively between 2000 and 2014, following our hospital protocol for ROP [13].

Chorionicity and zygosity were determined for the different twins. Zygosity of each twin pair was determined by ultrasound scan of the fetus or histopathological examination of the placenta.

Twin pairs were considered to be monozygotic if their placenta was monochorionic, regardless of whether it was monoamniotic or diamniotic. Dichorionic twins were considered dizygotic only if they had different blood groups or sex; otherwise they were excluded from the analysis [14]. Twins born as a result of in vitro fertilization were considered dizygotic.

The data were collected prospectively from the inclusion of premature infants in said protocol to the completion of the ophthalmologic monitoring, by medical discharge or death [15].

The degree of retinopathy in each eye studied was determined via indirect ophthalmoscopy with indentation and after pharmacological mydriasis by an expert ophthalmologist in this technique, using for this purpose a 20-dioptre lens. This lens provides a magnification of 2.5, allowing a 45° view of the retina, the equivalent of 8 papillary diameters [16, 17].

All the stages of ROP were included in the model. We verified that the samples of monozygotic and dizygotic twins to be studied did not present a significantly different sampling distribution with respect to potential confounding variables. For that purpose, we used parametric tests (analysis of variance and Student's *t*-test) and nonparametric tests (*χ* ^2^ and Fisher's exact tests) in order to analyze the continuous and dichotomous data, respectively.

The degree of correlation existing between the maximum stage of ROP in each pair of monozygotic and dizygotic twins was determined (right eye facing right eye). Once this correlation between monozygotic and dizygotic twins was calculated, we could determine the heritability. The concordance [18] of the stage of ROP in the right eye of both monozygotic and dizygotic twins was studied via Pearson correlation coefficient. For the final calculation of heritability, we used the formula proposed by Holzinger [19]. The difference between the stages of ROP in both eyes of the same premature infant determines the random error. The percentage unexplained by heritability, and by random error, is determined by environmental factors.

The presence or absence of ROP was considered a dependent variable. The predictive factors were gestational age of the premature infant, presence and degree of severity of hyaline membrane disease, patent ductus arteriosus, days of intubation, cesarean delivery, performance of transfusions, Apgar score at 1 minute and 5 minutes median at birth day, sepsis, cerebral hemorrhage development, X-ray CT/scan cerebral alterations, maternal hypertension, exchange transfusions, number of risk factors in the premature infant, maternal gestational diabetes, avascular retinal area, age of the mother, sex, birth weight (grams/100), and weight gain (grams/day) at 4–6 weeks.

The duration of oxygen was defined as the total number of days that the infant required the use of supplemental oxygen with mechanical ventilation (FiO2 > 21%), CPAP (>2 L), and nasal cannula (<2 L).

Sepsis was defined as a positive fungal or bacterial blood culture with the additional criterion of the presence of generalized infection symptoms and hematologic findings.

Avascular area was defined as a number of papillary diameters yet to be vascularized in the temporal area of the retina in the first study-related eye examination.

We calculated the weight gain rate (grams/day) as the weight gain between birth and the first study-related eye examination divided by days of life at first study-related eye examination (Table 1).

A binary logistic regression analysis was performed to determine predictive factors for the development of ROP, thus obtaining the best predictive model. In order to determine the existence of a correlation between ROP and the different variables registered at neonatal ICU for the total of 257 premature infants, a univariate and mixed-effects logistic regression (MELR) analysis was carried out using the statistical package SPSS 15.0 Inc., Chicago. The independent variables or risk factors considered in our study are described in Table 1. We first analyzed the predictors of ROP using univariate analysis followed by a multivariate logistic regression model. The final multivariate model retained the risk factors with *p* < 0.05. The odds ratio (OR) with 95% CIs associated with each predictor was calculated from logistic regression models.

## 3. Results

### 3.1. Comparison of Risk Factors between Monozygotic and Dizygotic Twins

The two cohorts of twins studied (monozygotic and dizygotic) have similar baseline clinical characteristics and no significant statistical differences were found between them (Table 1). Only the age of the mother was significantly higher in dizygotic twins than in monozygotic twins (*p* < 0.01).

No significant difference in the number of days with FiO2 > 21% was found in the monozygotic and dizygotic twins requiring controlled mechanical ventilation (*p* = 0.15), respiratory support (*p* = 0.47), and days of nasal cannula (*p* = 0.1) (Table 1). Maximum FiO2 was defined as the highest value of FiO2 obtained at any time while the preterm infants were intubated. The mean maximum FiO2 in the group of dizygotic twins was 55% ± 41% and in the monozygotic twins was 47% ± 42% (Student's *t*-test = 0.54, gl = 1, *p* = 0.058 n.s.). We found no significant differences in the mean maximum FiO2 in both groups.

Early sepsis was defined as sepsis developed within the first week of life and late sepsis as sepsis developed within 8 to 28 days. The dizygotic twins had early sepsis (*n* = 12) and late sepsis (*n* = 6), and the monozygotic twins had early sepsis (*n* = 6) and late sepsis (*n* = 8). We found no significant differences in the number of early sepsis (*χ* ^2^ = 0.006, df = 1, *p* = 0.94) or late sepsis (*χ* ^2^ = 2.37, df = 1, *p* = 0.12) in both groups.

The living premature with sepsis showed early failure of 2 to 4 organs, in dizygotic twins (*n* = 9) and in monozygotic ones (*n* = 7). We found no significant differences of organic failure in both groups (*χ* ^2^ = 0.14, df = 1, *p* = 0.72).

The lack of differences in the two samples studied allowed us to compare the heritability of both of them, reducing biases associated with variables that act as potential confounding factors.

### 3.2. Genotypic, Environmental, and Random Factors Explaining the Variability of ROP

Ideally, it can be hypothesized that a phenotypic character measured by a variable *t* is formed as the sum of three components [20]: the first one is the influence of the genetic variable *t* (*H*), or simply (*h*); the second one is the influence of the environmental variable (*A*), or simply (*a*); and the third one is the random residue (*ε*), so that

(1) $$\begin{array}{c} t=h+a+\varepsilon . \end{array}$$

In ROP, we proceed to break down and study the components (genetic, environmental, and random) of the described formula.

#### 3.2.1. The Influence of Genetics or Heritability (*h*)

We contrasted the degree of ROP between infants born as monozygotic twins and as dizygotic twins. For this purpose, we made a consecutive and paired comparison of the right eye of both twins. We studied the correlation of the stage of ROP in the two groups described. After this analysis, we obtained a correlation (*r*) of the degree of ROP in infants born from a multiple birth (dizygotic twins) of *r* = 0.4184 and a correlation of the degree of ROP in infants born from a multiple birth (monozygotic twins) of *r* = 0.8419.

Once the correlation values for the above groups were known, and aiming to determine the proportion of the degree of ROP exclusively attributable to the genetic (heritability) factor, we used the mathematical formula given by Holzinger [19]:

(2) $$\begin{array}{c} h=\frac{\left(r_{monozygotic}-r_{dizygotic}\right)}{\left(1-r_{dizygotic}\right)} \end{array}$$

in which *h* is heritability and *r* is correlation coefficient.

After assigning the value of the correlations of the above paragraphs to Holzinger's formula, we found that the degree of ROP is 72.82% determined exclusively by the genetics received from their parents.

#### 3.2.2. Random Residue (*ε*)

We consider that both right and left eyes of the premature infants should have the same degree of ROP since they are determined by the same genetic pattern (100%) and they are both under the influence of the same environmental factors.

To determine the* percentage of local variability in twins*, that is, the expected difference in the degree of ROP between the eyes of the same twin, a correlation analysis was carried out for this degree between the right and the left eye (intrapair correlation). Thus, a correlation of 95.88% (*r* ^2^ = 0.9588, *t*_exp_ = 50, gl = 1, *p* < 0.0001) was obtained and consequently, the local variability found would be 4.12% (100% − 95.88%). 4.12% of the eyes develop a higher or lesser degree of retinopathy than the contralateral eye.

#### 3.2.3. Shared (*a* ^1^) and Nonshared (*a* ^2^) Environmental Influence

Similarly, we can determine the portion of variability attributable to environmental influence (shared and nonshared variable) taking into account that

(3) $$\begin{array}{c} \text{Shared environmental influence}\left(\;a^{1}\;\right)=r_{m}-h, \end{array}$$

where *a* ^1^ = (0.8419 − 0.7282) = 0.1137 × 100 = 11.37%. Consider

(4) Nonshared environmental influencea2=1−h−a1−ε,

where *a* ^2^ = 1 − 0.7282 − 0.1137 − 0.0412 = 0.1169 × 100 = 11.69%.

**Figure 1:**
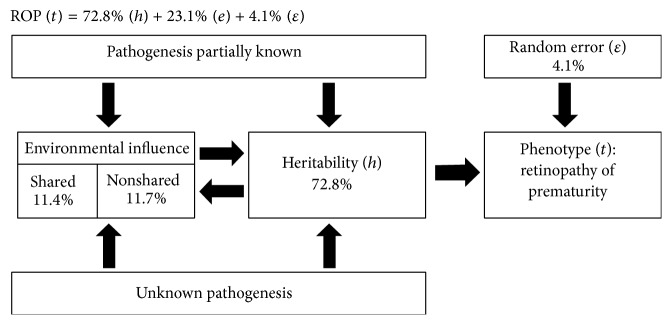
The pathophysiology of ROP is dynamic, influenced by genetic, environmental, and random factors. *ε*, random error; *t*, ROP phenotype; *h*, heritability; *e* = environmental factors.

In our case, and after completing the described calculations, the equation that explained the total variability of ROP would be the one given in Figure 1.

### 3.3. Binary Logistic Regression of the Predictive Model for the Presence or Absence of ROP and Environmental Factors without Genetic Component

In univariate analyses, the predictive factors associated with an increased risk of ROP were low gestational age (*p* < 0.0001), greater degree of hyaline membrane (*p* = 0.003), presence of ductus arteriosus (*p* = 0.001), greater number of days with CPAP (*p* = 0.002), greater number of days with nasal cannula (*p* = 0.016), greater number of transfusions (*p* < 0.0001), greater number of risk factors (*p* < 0.0001), presence of cerebral hemorrhage (*p* < 0.037), presence of brain lesions detected by CT (*p* < 0.0001), male (*p* < 0.015), and low birth weight (grams) (*p* < 0.0001). There was an increased risk of ROP in multiple birth versus single birth (*p* = 0.015), but there was no significant difference between monozygotic and dizygotic twins (*p* = 0.7).

Multiple stepwise regression analyses reliably revealed 3 independent risk factors for ROP (any grade): days of intubation (odds ratio (OR): 1.1 (CI: 1.05–1.16), *p* < 0.0001), weight gain (grams/day) (OR: 0.92 (CI: 0.87–0.96), *p* < 0.001), and sepsis (OR: 2.23 (CI: 1.18–4.22), *p* = 0.014). These significant predictors explained 32% of its variability (Table 2).

The weight is in grams (*p* = grams gained/day).

The risk is calculated with the following formula:

(5) Logit:−0.229+0.099 (days of intubation)−0.088 (weight gain grams/day)+0.802 (sepsis)Risk of ROP=11+e−logit.

In the environmental variables we can observe that the days of intubation and the degree of sepsis act as risk factors, while the postnatal weight gain acts as a protective factor.

The prediction of ROP was evaluated using the area under the receiver operating characteristic curve (AUC). Days of intubation predicted ROP with an AUC of 0.724 (95% CI: 0.66–79). When the weight gain was included, the AUC improved slightly to 0.729 (95% CI: 0.66–0.80). Finally, when sepsis was added, the AUC also improved slightly to 0.731 (95% CI: 0.66–0.802). We found that the intubation days for mechanical ventilation with FiO2 > 21%, at the first study-related eye examination, were an important predictor for ROP.

## 4. Discussion

ROP is an abnormal vascular proliferative retinopathy that occurs between 30 and 45 weeks of postgestational age. Its appearance and development are conditioned by the joint action of genetic [21, 22], environmental, epigenetic, and random factors [11, 12].

In our series, monozygotic twins presented the same characteristics and risk factors as dizygotic twins. Only the age of the mother was higher in the group of dizygotic twins, a fact which was also found in other series [23].

The appropriate development of the fetal retina and brain requires optimum connections between millions of neurons. Despite the highly complex nature of this process, neurons in the brain establish the appropriate connection with a low error margin (3%) [24]. Both eyes of the premature infant have the same genetic basis, share general epigenetic changes, and are influenced by the same environmental factors. Nevertheless, we found a difference of 4.1% in the stage of ROP between both eyes. This value, attributed to the* random error*, may be explained by the highly complex nature of the neural and vascular development of the retina.

Heritability could be defined as the portion of phenotypic variation associated with genetic variation. Twin studies allow us to obtain the heritability rate. In the specific case of bronchopulmonary dysplasia, the estimated heritability would vary between 79% and 82% [25]. Regarding patent ductus arteriosus, the heritability would be 76.1% [26] and in the case of apnea of prematurity 87% [14]. The heritability of ROP was estimated at 72.8% in our study, similar to that provided by Bizarro, which estimates 70.1% [9]. The neurovascular development of the retina is, to a large extent, genetically conditioned [27] and it is modified by a great number of genes [27] and cytokines [28, 29] that oscillate depending on the local and general environmental conditions. These factors act as “switches” activating or deactivating those genes upon which they exert their influence, therefore modulating the structure and the expression rate of the genetic code [11, 30–34].

The entire human genome consists of 20,000 genes. In patients with ROP, known mutations in 4 genes—Norrin (NDP), BDNF variants (development factor associated with the brain), low density lipoprotein receptor-related protein (LRP5), and frizzled family receptors 4 (FZD4)—aggravate this disease but they are rare [1]. Nevertheless, the study of patients with ROP shows alterations of almost 15% of the genome during the first month of life [35]. The hypoxia-reoxygenation phenomena determine protective responses in eye tissues such as glycolysis, vasodilatation, angiogenesis, and induced transcription of erythropoietin. This can produce transcriptional responses of the eyes affecting the inflammation, angiogenesis, energy failure, and RAS system [35, 36].

The intense retinal neurovascular coupling determines that vascular networks are correlated with neural development. In the first stage of ROP, suppression of development factors occurs due to hyperoxia and loss of fetal-maternal interaction. In the second stage, hypoxia, ischemia, and inflammation of the retina favor vascular proliferation. In our study, modifiable environmental variables with a higher risk of ROP include number of days of intubation [37, 38] (indirect indicator of hyperoxia), postnatal low weight gain (indirect indicator of insufficient nutrition), and development of sepsis (indirect indicator of inflammation).

In conclusion, in this study we break down and establish, for the first time, specific values for each factor that mediate and determine the total variability attributable to ROP: 72.8% is attributable to genetics, 11.4% to shared environmental conditions, and 11.7% to nonshared environmental conditions, and 4.1% is generated by random residue. Environmental variables that best explain the risk of ROP are higher number of days of intubation, sepsis, and postnatal low weight gain. Although there is a strong heritable predisposition in ROP, it would be the joint action of these three environmental factors, partly modifiable, which could reduce the risk of ROP.

## Figures and Tables

**Table 1:** Risk factors for the development of ROP were comparable between monozygotic and dizygotic twins.

Variable	Monozygotics	Dizygotics	*p* value
	(*n* = 38)	(*n* = 66)	
Gestational age (weeks)	30.31 ± 1.3	30.24 ± 1.9	*p* = 0.81
Degree of hyaline membrane			
Degree 0	8 (21%)	16 (24.2%)	*p* = 0.96
Degree I	5 (13.2%)	6 (9.1%)	*p* = 0.63
Degree II	12 (31.6%)	16 (24.1%)	*p* = 0.56
Degree III	9 (23.7%)	24 (36.4%)	*p* = 0.26
Degree IV	4 (10.5%)	4 (6.1%)	*p* = 0.66
Ductus arteriosus	4 (10.5%)	9 (13.6%)	*p* = 0.87
Days of intubation	7.9 ± 10.1	5.2 ± 7.9	*p* = 0.15
Attendance days with CPAP	4.8 ± 4	5.4 ± 5	*p* = 0.47
Attendance days with nasal cannula	3.5 ± 4.9	5.9 ± 9.9	*p* = 0.1
Cesarean birth (yes/no)	34 (89.5%)	61 (92.4%)	*p* = 0.87
Transfusions (yes/no)	22 (57.9%)	28 (42.4%)	*p* = 0.18
Apgar at 1 minute	6.1 ± 3	6.2 ± 2.3	*p* = 0.84
Apgar at 5 minutes	6.9 ± 3.9	7.8 ± 2.6	*p* = 0.19
Number of risk factors	7.6 ± 2.2	7.6 ± 2.9	*p* = 0.99
Sepsis (yes/no)	14 (36.8%)	18 (27.3%)	*p* = 0.42
Cerebral hemorrhage (yes/no)	3 (7.9%)	10 (15.1%)	*p* = 0.44
X-ray CT/scan alterations (yes/no)	7 (18.4%)	14 (21.2%)	*p* = 0.98
Maternal HTN (yes/no)	8 (21%)	10 (14.7%)	*p* = 0.61
Exchange transfusion (yes/no)	6 (15.8%)	8 (12.1%)	*p* = 0.81
Avascular area (papillary diameter)	2.1 ± 1.7	2.4 ± 2.15	*p* = 0.46
Age of the mother	30.2 ± 5.5	33.1 ± 4.5	*p* < 0.01
Sex (male/female)	20 (52.6%)	30 (45.4%)	*p* = 0.61
Weight gain (gr/day) 4–6 weeks	12.4 ± 8.1	12.6 ± 6.4	*p* = 0.91
Birth weight (gr/100)	12.3 ± 2.5	12.8 ± 2.9	*p* = 0.41
Retinopathy (yes/no)	15 (39.5%)	30 (45.4%)	*p* = 0.69

**Table 2:** Multivariate analysis for predictors of ROP. OR, odds ratio; CI, confidence interval.

Predictors	Number of infants at risk	Number of ROP (%)	Adjusted OR (95% CI)	*p* value
Days of intubation				
0	108	23 (21.3)	1 (reference)	
1–3	49	11 (22.4)	1.07 (0.43–2.56)	0.9
4–9	46	20 (44.4)	2.84 (1.27–6.39)	0.008
>9	54	39 (72.2)	9.6 (4.26–22.01)	<0.001
Weight gain				
>17	68	11 (16.7)	1 (reference)	
≥12 <17	72	21 (29.2)	2.39 (0.96–6.04)	0.06
≥9 <12	49	18 (36.7)	3 (1.16–7.86)	0.02
<9	68	43 (63.2)	8.9 (3.69–21.92)	<0.001
Sepsis				
No	179	51 (28.5)	1 (reference)	
Yes	79	42 (53.8)	2.9 (1.62–5.27)	<0.001
